# Robotic-Assisted Repair of Morgagni Hernia in an Adult Patient: A Case Report

**DOI:** 10.7759/cureus.114385

**Published:** 2026-08-11

**Authors:** Ermer I Guevara-Elizondo, Andrea Cantú Amaya, Gustavo Cervantes Millán, Jorge González Acosta

**Affiliations:** 1 Department of General Surgery, Centro Médico ABC, Mexico City, MEX; 2 Department of General Surgery, Tecnológico de Monterrey, Mexico City, MEX

**Keywords:** diaphragmatic hernia, minimally invasive surgery, morgagni hernia, robotic hernia repair, robotic surgery

## Abstract

Morgagni hernia is a rare congenital diaphragmatic defect representing a small percentage of all diaphragmatic hernias. Although often diagnosed in childhood, many cases remain asymptomatic until adulthood and are frequently identified incidentally or during the evaluation of nonspecific respiratory or gastrointestinal symptoms. Surgical repair is generally recommended because of the potential risk of incarceration and strangulation. Minimally invasive approaches have become widely adopted treatment strategies, and robotic-assisted surgery has emerged as an alternative that may offer technical advantages in complex diaphragmatic repairs. We present the case of an adult patient with an anterior diaphragmatic hernia who underwent successful robotic-assisted repair with primary closure and mesh reinforcement. The procedure was completed without major complications, and postoperative recovery was favorable. This case highlights the feasibility of robotic-assisted Morgagni hernia repair and illustrates its potential role as a minimally invasive option for managing complex diaphragmatic defects.

## Introduction

Morgagni hernias represent a rare type of congenital diaphragmatic hernia, accounting for approximately 2%-4% of all congenital diaphragmatic hernias [1]. Their development is associated with incomplete fusion between the septum transversum and the muscular fibers of the diaphragm in the retrosternal region, resulting in a defect within the sternocostal triangle, also known as the foramen of Morgagni or the triangle of Larrey [2]. Although these hernias are commonly diagnosed during childhood, a significant proportion remain asymptomatic and may present during adulthood, usually with nonspecific symptoms such as abdominal pain or dyspnea, or as incidental findings on imaging studies [3].

Surgical repair is generally considered the treatment of choice, even in asymptomatic patients, because of the risk of serious complications such as incarceration or bowel strangulation [4]. Historically, open approaches through laparotomy or thoracotomy were most commonly employed. However, with advances in minimally invasive surgery, laparoscopy has become a preferred approach because of its lower morbidity, improved visualization of the defect, and faster postoperative recovery [5]. Nevertheless, laparoscopic repair presents technical limitations, particularly regarding intracorporeal suturing and mesh placement in the retrosternal region, prompting the use of alternative techniques [6].

In this context, robotic surgery has emerged as a feasible and safe minimally invasive option for the repair of Morgagni hernias in adults. Several reports have described potential technical advantages over conventional laparoscopy, including improved surgeon ergonomics, 3D visualization, and wristed instrumentation that facilitates precise retrosternal dissection and intracorporeal suturing in confined spaces [7,8]. These characteristics may be particularly advantageous when performing tension-free closure of large or complex diaphragmatic defects. Available reports have also described favorable perioperative outcomes, including low morbidity and rapid postoperative recovery; however, current evidence remains limited to case reports and small case series [9].

The use of mesh in Morgagni hernia repair remains a matter of debate. While primary closure may be considered for small defects, mesh reinforcement is often considered for larger defects to reduce the risk of recurrence [10]. Various mesh materials have been reported in the literature, including polypropylene, polytetrafluoroethylene (PTFE), composite, and absorbable biologic meshes, with variable outcomes regarding complications and recurrence rates [11]. In robotic surgery, extraperitoneal mesh placement may offer advantages by reducing direct contact between the mesh and abdominal viscera and potentially reducing visceral adhesions [12].

Although current evidence is primarily derived from case reports and small case series, available findings suggest that robotic Morgagni hernia repair is feasible and safe, with outcomes that appear comparable to those of laparoscopic surgery, and that it may offer additional advantages in selected cases [13,14]. Nevertheless, the cost and availability of robotic technology remain important limitations in many institutions [15].

We present the case of an adult patient diagnosed with a Morgagni hernia who underwent robotic-assisted repair using the da Vinci Surgical System (Intuitive Surgical, Inc., Sunnyvale, California, USA), accompanied by a critical review of the literature comparing minimally invasive techniques, with particular emphasis on differences between laparoscopic and robotic approaches regarding patient selection, surgical technique, mesh utilization, complications, and outcomes.

## Case presentation

**Figure 1:**
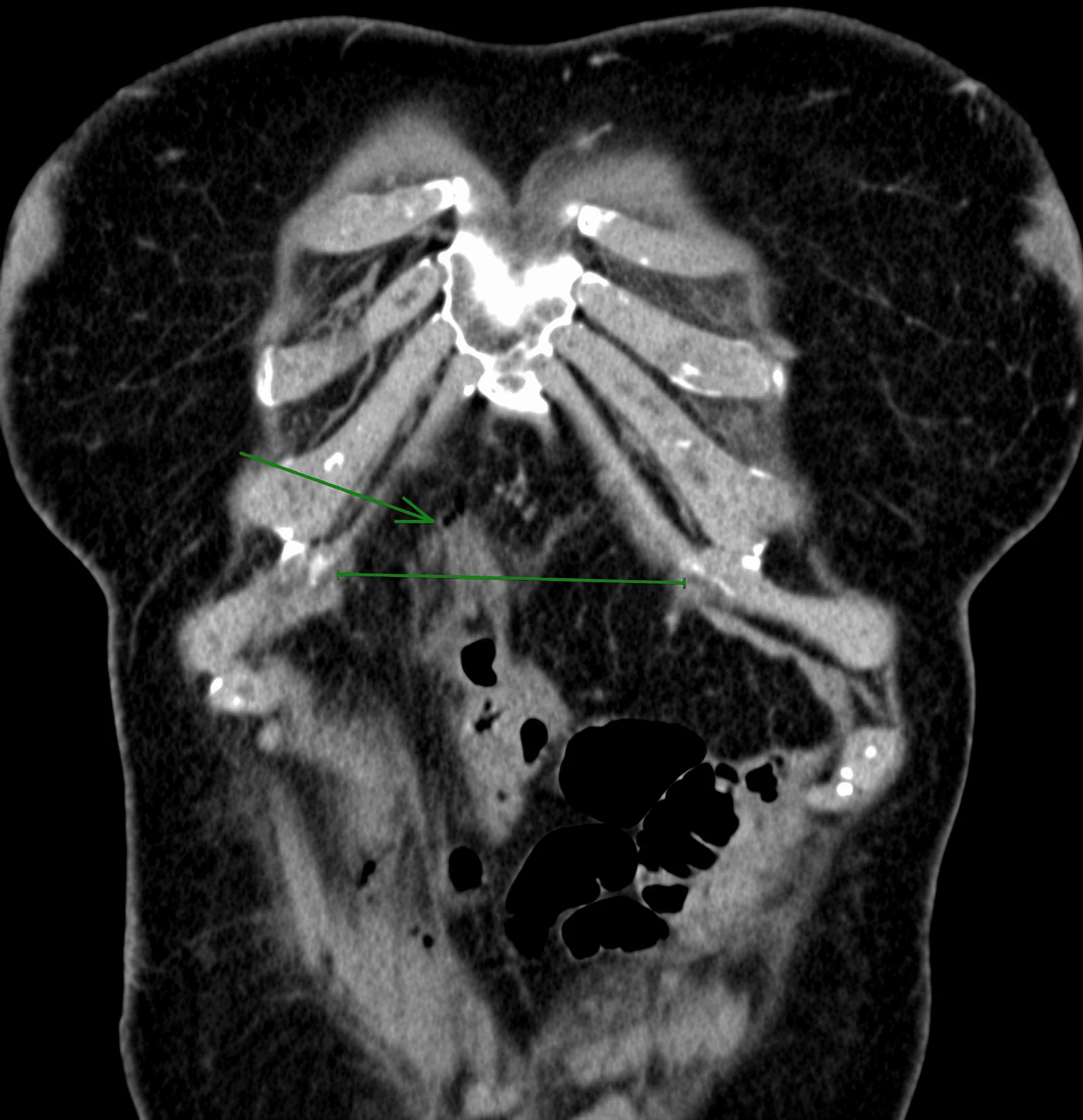
CT of the chest and abdomen. Anterior paramedian retrosternal diaphragmatic defect measuring up to 91 mm, with herniation of adipose tissue and transverse colon.

A 67-year-old female patient with a medical history significant for hypothyroidism and rheumatoid arthritis, both under medical treatment, was evaluated. During a laparoscopic cholecystectomy performed one month earlier, an anterior diaphragmatic hernia was incidentally identified. Subsequent CT of the chest and abdomen confirmed the presence of an approximately 91-mm anterior paramedian retrosternal diaphragmatic defect, with herniation of omental fat and the transverse colon into the thoracic cavity and no evidence of strangulation or vascular compromise. The patient was hemodynamically stable and scheduled for robot-assisted repair of the defect (Figure 1).

The procedure was performed under balanced general anesthesia, with the patient in the supine position with a reverse Trendelenburg tilt. Pneumoperitoneum was established using the Hasson technique through a 12-mm supraumbilical optical port, with the intra-abdominal pressure maintained at 12 mmHg. Under direct visualization, two 8-mm robotic working ports were placed at the level of the right and left midclavicular lines, approximately 2 cm above the umbilical plane. A 10-mm assistant port was inserted in the left flank, approximately 5-8 cm lateral and slightly caudal to the left robotic port. The da Vinci Surgical System (Intuitive Surgical, Inc., Sunnyvale, California, USA) was then docked with the boom oriented cephalad. A Nathanson liver retractor was inserted through a separate 5-mm subxiphoid incision to provide adequate liver retraction.

**Figure 2:**
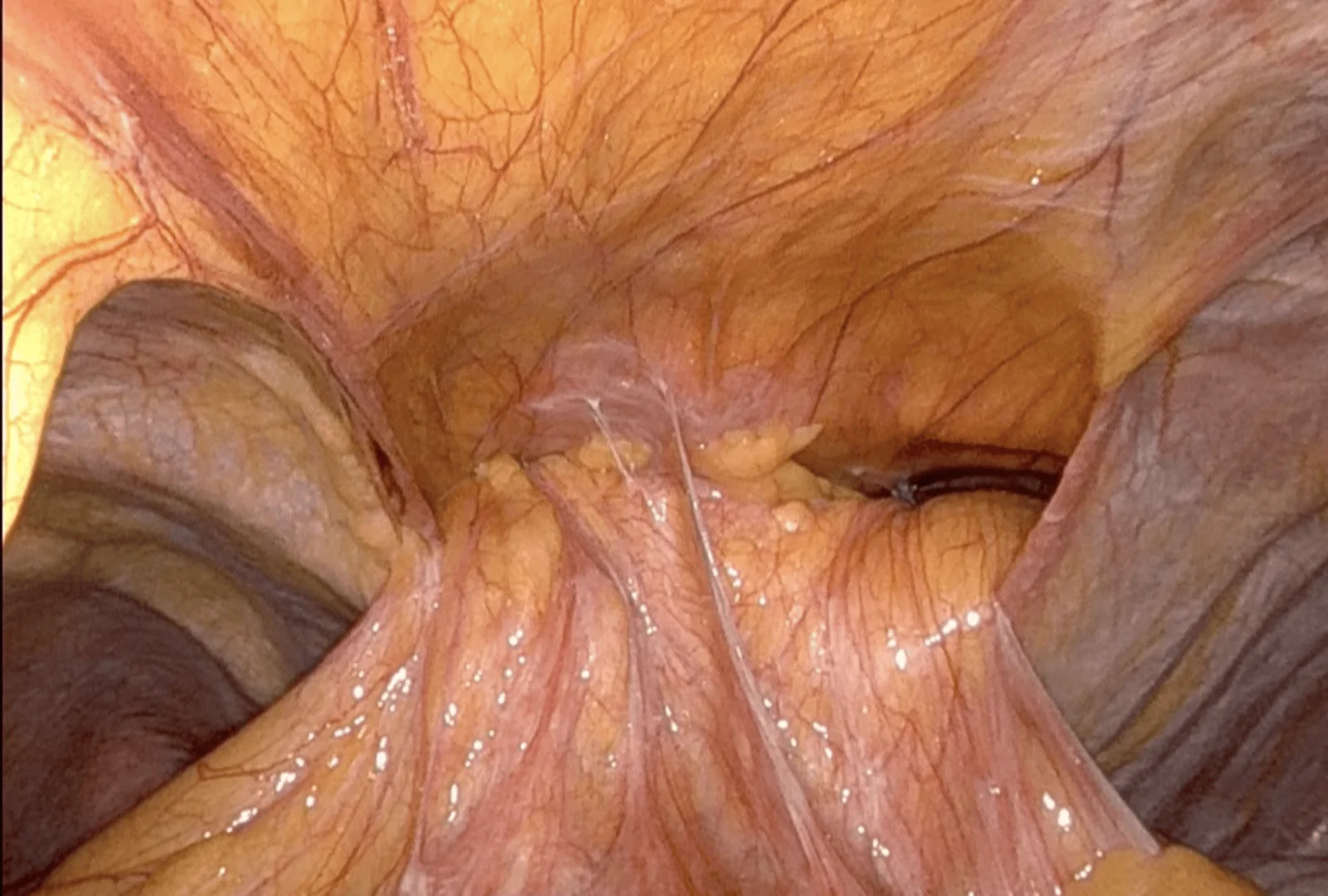
Morgagni hernia. Hernia contents consisting of the greater omentum and the round ligament of the liver.

Upon exploration of the abdominal cavity, a 9 × 10-cm retrosternal defect was identified. Although preoperative CT imaging had demonstrated herniation of the transverse colon into the thoracic cavity, intraoperative findings revealed spontaneous reduction of the bowel into the abdominal cavity. No evidence of ischemia, strangulation, or vascular compromise of the intestinal loops was observed. At the time of surgery, the hernia contents consisted only of the greater omentum and the round ligament of the liver (Figure 2). Complete atraumatic reduction of the remaining hernia contents was performed, and bowel viability was confirmed.

**Figure 3:**
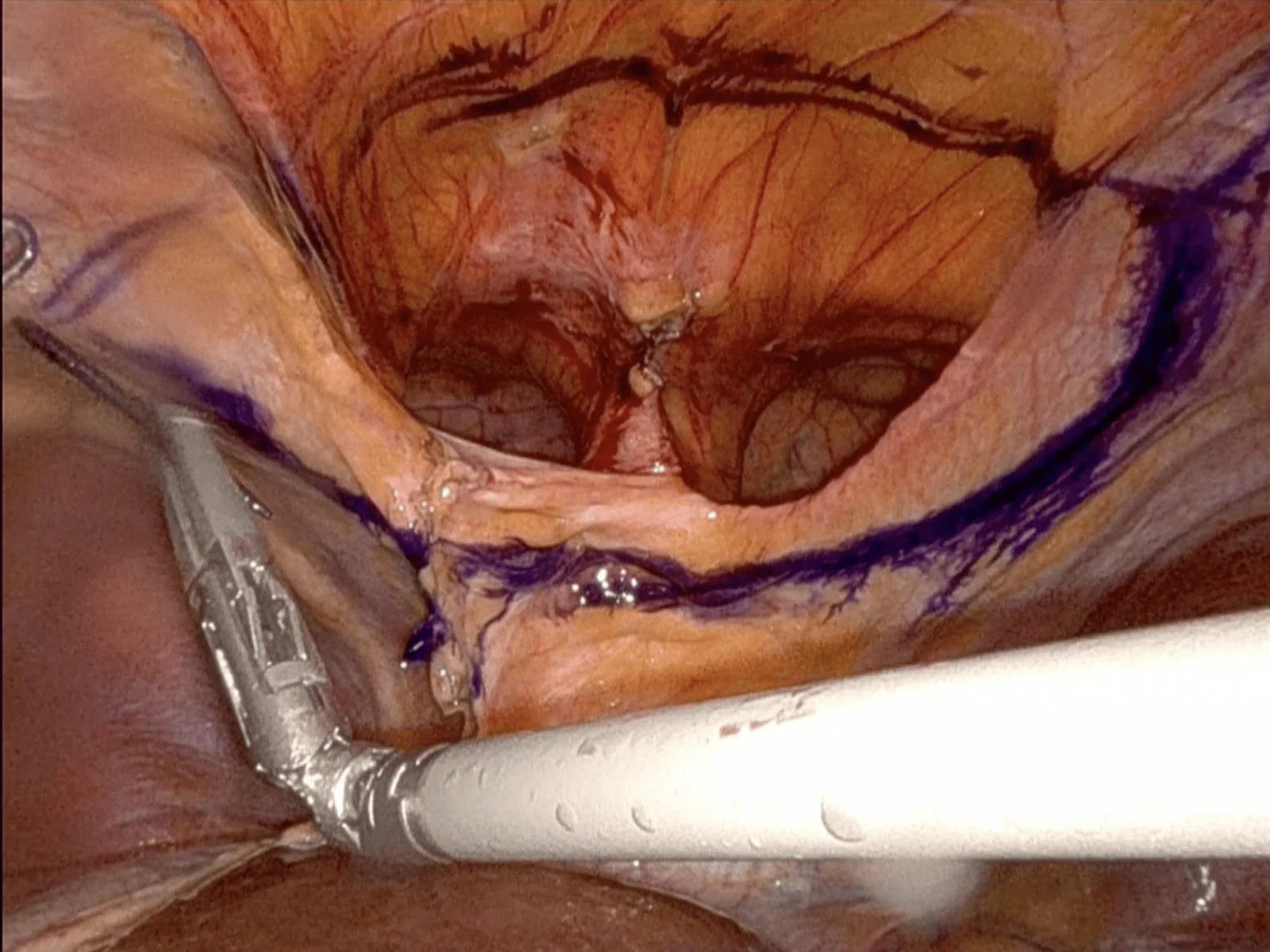
Intraoperative delineation of the hernia defect. Surgical ink marking outlining the margins of the hernia defect.

**Figure 4:**
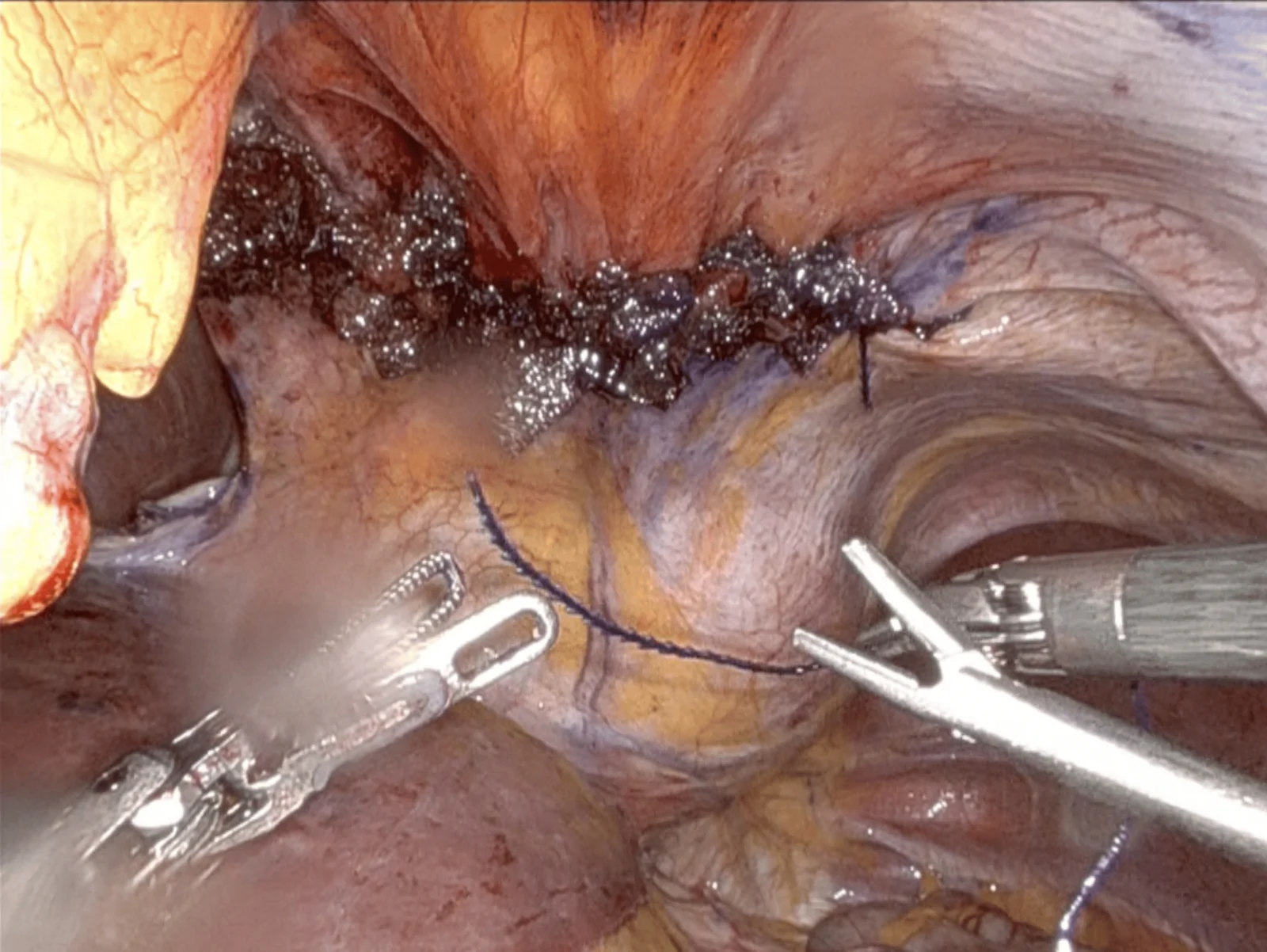
Primary hernia defect closure. Primary closure of the hernia defect using a barbed polydioxanone suture (Stratafix PDS Plus; Ethicon US, LLC, Raritan, New Jersey, USA) in a horizontal mattress configuration, reinforced with polytetrafluoroethylene (PTFE) pledgets (Ethicon US, LLC, Raritan, New Jersey, USA).

Following complete reduction of the hernia contents, the redundant lateral portion of the hernia sac was closed with continuous 2-0 polyglactin sutures (Vicryl®; Ethicon US, LLC, Raritan, New Jersey, USA) to facilitate tissue management. Attention was then directed to the diaphragmatic defect, which was outlined with surgical ink (Figure 3) and closed primarily using a barbed polydioxanone suture (Stratafix PDS Plus; Ethicon US, LLC, Raritan, New Jersey, USA), with PTFE pledgets (Ethicon US, LLC, Raritan, New Jersey, USA) placed using a horizontal mattress technique (Figure 4). Complete tension-free approximation of the diaphragmatic edges was achieved before reinforcement with a bioresorbable synthetic mesh.

**Figure 5:**
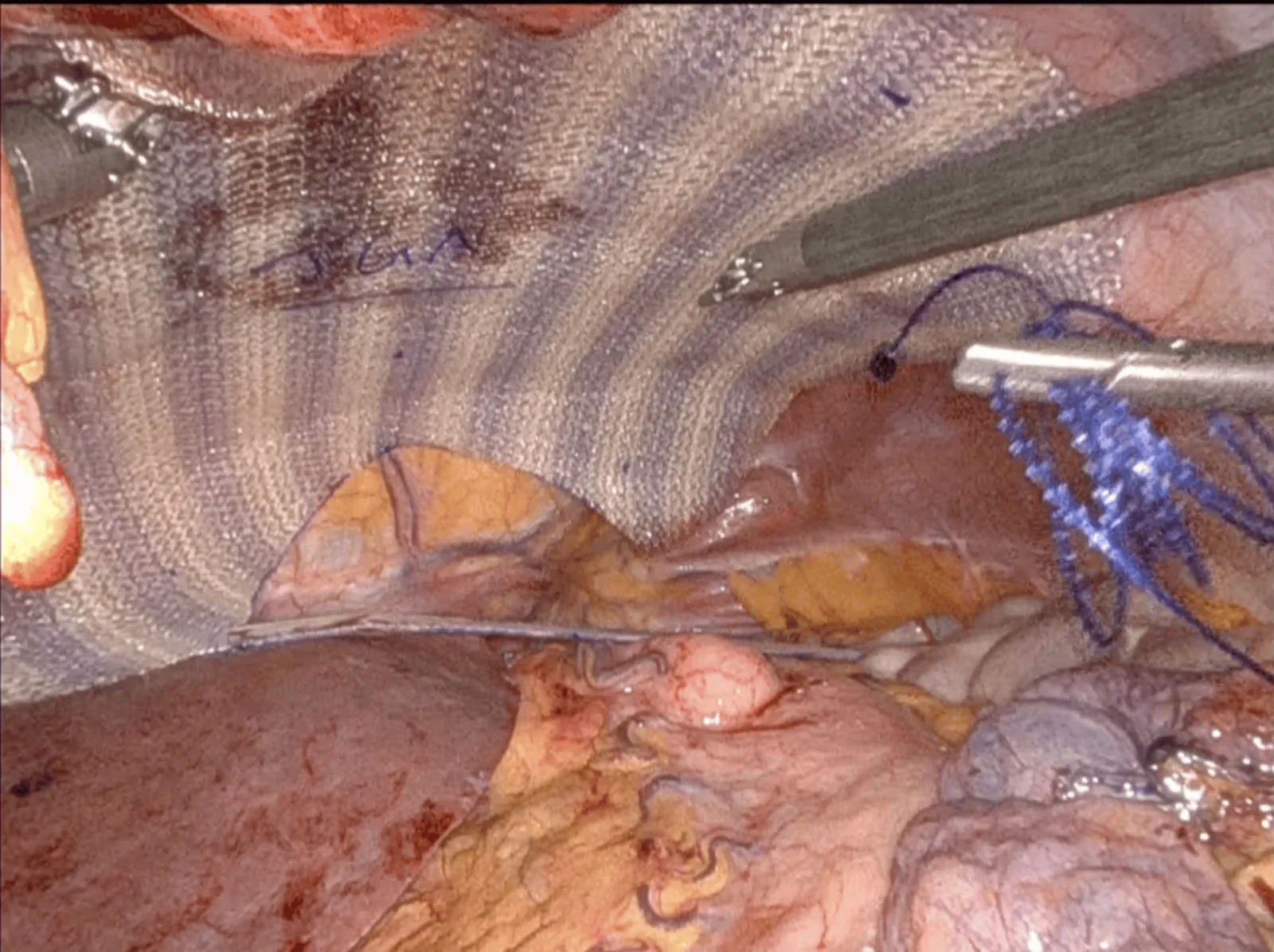
Bioresorbable synthetic mesh placement. Placement of a 12 × 15-cm bioresorbable synthetic mesh (Phasix™ ST Mesh; Becton, Dickinson and Company, Franklin Lakes, New Jersey, USA) to reinforce the diaphragmatic repair.

The pleura was carefully preserved throughout the dissection to avoid iatrogenic pneumothorax. A 12 × 15-cm bioresorbable synthetic mesh (Phasix™ ST Mesh; Becton, Dickinson and Company, Franklin Lakes, New Jersey, USA) was then placed to reinforce the diaphragmatic repair (Figure 5).

The procedure was completed without complications, with minimal blood loss (5 mL) and a total operative time of 3 hours and 45 minutes.

The patient was extubated in the operating room and transferred to the recovery unit in stable condition. During the immediate postoperative period, she reported mild pain (3/10), without signs of peritoneal irritation or respiratory compromise. Follow-up chest radiography demonstrated bibasal atelectasis and cervical subcutaneous emphysema, without evidence of pneumothorax or other major complications.

On postoperative day one, the patient remained hemodynamically stable, with adequate pain control under multimodal analgesia and antibiotic coverage with ceftriaxone. Postoperative CT demonstrated complete repair of the previously identified Morgagni hernia, with no residual herniation through the anterior retrosternal diaphragmatic defect. Expected postoperative changes included increased density of the surrounding fat and small air collections adjacent to the repair site, consistent with recent diaphragmatic repair and mesh placement. No early evidence of recurrent herniation or mesh-related complications was identified.

At the three-month follow-up, the patient remained clinically well, with no evidence of hernia recurrence or GI symptoms. She reported mild residual discomfort localized to the xiphoid region at the site of mesh fixation, which was adequately controlled with conservative measures and did not interfere with her daily activities. The patient denied symptoms of gastroesophageal reflux, dysphagia, respiratory complaints, or limitations in functional status.

At the time of writing, the patient remains under scheduled clinical follow-up, with surveillance planned through 12 months and imaging reserved for recurrent symptoms or suspected recurrence.

## Discussion

Morgagni hernia in adults remains a surgical challenge because of its rarity and the absence of standardized management guidelines. Minimally invasive approaches have progressively replaced open surgery, with laparoscopy becoming a widely preferred approach [1]. However, the introduction of robotic surgery has created new technical possibilities that warrant comparative evaluation.

The laparoscopic approach provides excellent exposure of the retrosternal defect and is associated with shorter hospital stays and faster recovery compared with open surgery [1,2]. However, it presents technical challenges related to intracorporeal suturing and mesh placement, particularly in large defects or those located near the pericardium [3].

In this context, the robotic approach represents a promising minimally invasive alternative for selected patients with large or technically complex Morgagni hernias. Reported advantages include improved surgeon ergonomics, 3D visualization, and greater precision during retrosternal dissection and intracorporeal suturing [4,5]. The robotic platform may also facilitate preperitoneal or extraperitoneal mesh placement in selected cases. However, in the present case, after intraoperative assessment, preperitoneal mesh placement was considered but not pursued because the planned reconstruction involved intraperitoneal reinforcement with a bioresorbable synthetic mesh. Current evidence remains limited to case reports and small institutional series, and larger comparative studies with long-term follow-up are needed to better define the role of robotic surgery relative to conventional laparoscopy [5-7].

Primary closure may be considered for small defects when a tension-free repair can be achieved [1]. For larger defects, mesh reinforcement is often recommended to reduce the risk of recurrence [2,3].

Different prosthetic materials, including permanent synthetic, composite, biologic, and bioresorbable synthetic meshes, have been described for reinforcement after primary closure. However, the optimal prosthetic choice remains controversial, as comparative long-term evidence regarding recurrence, tissue incorporation, and mesh-related complications remains limited [2,4,6].

Reported complications include recurrence, seroma formation, pneumothorax, and postoperative pain [1,2]. Although recurrence is uncommon following laparoscopic repair, it has been reported after primary closure without mesh reinforcement [3]. Seroma formation and pneumothorax are infrequent but recognized complications, particularly when the hernia sac is resected [2].

Robotic repair has been associated with low complication rates, limited postoperative pain, and rapid recovery [4,7]. Nevertheless, increased costs and potentially longer operative times, particularly during the initial learning curve, remain important limitations [5].

Current literature describes several potential advantages of robotic surgery, including greater precision during intracorporeal suturing [4,5], limited postoperative pain and rapid recovery [7], and the ability to place mesh in an extraperitoneal position with minimal fixation [6]. These technical characteristics may make robotic repair particularly useful in selected patients with large or complex defects requiring precise dissection and intracorporeal suturing.

Published reports of robotic Morgagni hernia repair have described potential clinical and technical advantages, primarily related to visualization and intracorporeal suturing. Kunitsky A et al. described successful robotic repair in a 77-year-old patient, with discharge on postoperative day one, highlighting the visualization and dexterity offered by the robotic platform. Similarly, Arevalo G et al. reported technically successful robotic primary closure in three adult patients; two were discharged on postoperative day one and one on postoperative day three. Koza D et al. reported a low complication rate and no recurrence or chronic pain during a median follow-up of 284 days in a series of seven patients [7,15,16].

Further expanding on these intraoperative applications, Bertram SC et al. reported that single-stage simultaneous robotic repair of inguinal and Morgagni hernias was feasible and suggested that this approach may reduce the number of exposures to general anesthesia and surgeon fatigue. Huang YJ and Fang YL reported robot-assisted repair in two adults with congenital diaphragmatic hernias, including one patient with a Morgagni hernia, and highlighted the benefits of 3D visualization and precise suturing. No complications or recurrence were reported in their small case series (Table 1) [17,18].

**Table 1:** Published reports of robotic Morgagni hernia repair. Summary of published cases and case series of robotic Morgagni hernia repair, including defect characteristics, surgical techniques, mesh utilization, outcomes, complications, and reported recurrence.

Article/authors	Defect size	Technique used	Mesh used	Advantages	Complications	Recurrence
Kunitsky A et al. [7]	3 cm in diameter	Robotic repair with plication of the hernia sac, transverse approximation of the diaphragm, and mesh placement	9-cm round polyester mesh (Symbotex; Medtronic, Minneapolis, Minnesota, USA)	Minimally invasive approach considered ideal for elderly patients; excellent visualization and dexterity; rapid recovery, with discharge on postoperative day 1	None; pneumothorax was ruled out by postoperative chest radiography	No recurrence reported [7]
Arevalo G et al. [15]	Not specified in centimeters for the three cases described	Robotic repair involving reduction of the hernia contents, primary closure of the defect, and mesh reinforcement	Mesh with a bioabsorbable coating in two patients and biologic mesh in one patient	Allows precise dissection of the sac and tension-free primary repair; short hospital stay, with two patients discharged on postoperative day 1	Serosal tear of the transverse colon during reduction in one patient, repaired intraoperatively; no postoperative complications	No recurrence reported [15]
Koza D et al. [16]	Median length of 8 cm and width of 4 cm (IQR: length, 6-11 cm; width, 4-10 cm)	Robotic approach using an isolated transabdominal preperitoneal (TAPP) technique (42.9%) or combined with transversus abdominis release (TAR) for simultaneous incisional hernias (57.1%)	Macroporous midweight polypropylene mesh	Highly precise anatomical dissection, low overall complication rate, and excellent surgical outcomes	Immediate postoperative pain in 42.9% of patients (three cases) and one seroma (14.3%) that resolved spontaneously	No recurrence during a median follow-up of 284 days [16]
Bertram SC et al. [17]	5-cm right-sided Morgagni hernia	Single-stage simultaneous robotic repair of an inguinal hernia and the Morgagni hernia; primary diaphragmatic closure with barbed suture and mesh reinforcement	Porcine biologic mesh	Feasible and safe technique; reduces surgeon fatigue and avoids subjecting the patient to multiple episodes of general anesthesia; complete resolution of dyspnea and reflux	No perioperative complications reported	No recurrence mentioned during postoperative follow-up [17]
Huang YJ and Fang YL [18]	5 × 6 cm in the Morgagni case involving a 72-year-old patient	Robotic-assisted primary repair using nonabsorbable interrupted sutures; direct primary closure without mesh	None (primary suture repair)	3D magnification provides excellent visualization in deep spaces, facilitating precise and safe suturing of the diaphragm; discharge on postoperative day 4	No intraoperative or postoperative complications	No evidence of recurrence during the five months follow-up period [18].

Our case differs from previously reported robotic repairs because, despite the diaphragmatic defect measuring approximately 91 mm, tension-free primary closure was achieved and subsequently reinforced with a bioresorbable synthetic mesh. The postoperative course was favorable and without major complications despite the patient’s comorbidities, further supporting the technical feasibility of robotic-assisted repair in selected complex adult presentations.

## Conclusions

Morgagni hernia in adults is a rare condition that is often diagnosed incidentally during imaging studies or unrelated surgical procedures. Despite its frequently asymptomatic presentation, surgical repair is generally recommended because of the potential risk of serious complications, including incarceration and strangulation. Minimally invasive surgery has become the preferred treatment strategy, with laparoscopy widely used because of its favorable postoperative outcomes and lower morbidity compared with open surgery. However, technical challenges related to intracorporeal suturing and mesh placement in the retrosternal space may complicate its use in more complex cases.

Robotic-assisted repair has been reported as a safe and feasible minimally invasive alternative that may provide technical advantages in selected cases, including enhanced 3D visualization, improved ergonomics, and greater precision during intracorporeal suturing. These advantages may facilitate the repair of large or technically challenging diaphragmatic defects. Although extraperitoneal mesh placement has been described as a potential advantage of the robotic platform, the present case involved intraperitoneal reinforcement with a bioresorbable synthetic mesh after successful primary closure. Current evidence remains limited to case reports and small case series; therefore, further studies involving larger patient populations and longer follow-up periods are required to better define the role of robotic surgery in the management of adult Morgagni hernias.
